# Effects of Dietary Arginine and Glutamine on Alleviating the Impairment Induced by Deoxynivalenol Stress and Immune Relevant Cytokines in Growing Pigs

**DOI:** 10.1371/journal.pone.0069502

**Published:** 2013-07-29

**Authors:** Li Wu, Wence Wang, Kang Yao, Ting Zhou, Jie Yin, Tiejun Li, Lin Yang, Liuqin He, Xiaojian Yang, Hongfu Zhang, Qi Wang, Ruilin Huang, Yulong Yin

**Affiliations:** 1 Research Center of Healthy Breeding of Livestock and Poultry, Hunan Engineering and Research Center of Animal and Poultry Science, and Key Laboratory of Agro-Ecological Processes in Subtropical Region, Institute of Subtropical Agriculture, Chinese Academy of Sciences, Changsha, Hunan, China; 2 College of Animal Science, South China Agricultural University, Guangzhou, China; 3 Guelph Food Research Centre, Agriculture and Agri-Food Canada, Guelph, Ontario, Canada; 4 China National Key Laboratory of Animal Nutrition, Beijing Animal and Veterinary Science Institute, Chinese Agricultural Academy, Beijing, People’s Republic of China; National Institute of Agronomic Research, France

## Abstract

Deoxynivalenol (DON) is a mycotoxin that reduces feed intake and animal performance, especially in swine. Arginine and glutamine play important roles in swine nutrition. The objective of this study was to determine the effects of dietary supplementation with arginine and glutamine on both the impairment induced by DON stress and immune relevant cytokines in growing pigs. A total of forty 60-d-old healthy growing pigs with a mean body weight of 16.28±1.54 kg were randomly divided into 5 groups, and assigned to 3 amino acid treatments fed 1.0% arginine (Arg), 1.0% glutamine (Gln) and 0.5% Arg+0.5% Gln, respectively, plus a toxin control and a non-toxin control. Pigs in the 3 amino acid treatments were fed the corresponding amino acids, and those in non-toxin control and toxin control were fed commercial diet with 1.64% Alanine as isonitrogenous control for 7 days. The toxin control and amino acid treatments were then challenged by feeding DON-contaminated diet with a final DON concentration of 6 mg/kg of diet for 21 days. No significant differences were observed between toxin control and the amino acid groups with regard to the average daily gain (ADG), although the values for average daily feed intake (ADFI) in the amino acid groups were significantly higher than that in toxin control (P<0.01). The relative liver weight in toxin control was significantly greater than those in non-toxin control, arginine and Arg+Glu groups (P<0.01), but there were no significant differences in other organs. With regard to serum biochemistry, the values of BUN, ALP, ALT and AST in the amino acid groups were lower than those in toxin control. IGF1, GH and SOD in the amino acid groups were significantly higher than those in toxin control (P<0.01). The IL-2 and TNFα values in the amino acid groups were similar to those in non-toxin control, and significantly lower than those in toxin control (P<0.01). These results showed the effects of dietary supplementation with arginine and glutamine on alleviating the impairment induced by DON stress and immune relevant cytokines in growing pigs.

## Introduction

Mycotoxins are toxic fungal secondary metabolites that are found worldwide in various foods and animal feeds. Deoxynivalenol (DON), also known as vomitoxin, is a kind of mycotoxin that is mainly produced by *Fusarium graminearum*, which is found in grains, such as barley, maize and wheat [1], [2]. Contamination with DON will cause fusarium head blight (FHB) in grains and create a food-safety risk in human and animal food chains [3]. DON inhibits DNA, RNA and protein synthesis, as well as mitochondrial function in eukaryotic cells. It exerts an immunosuppressive effect in humans and animals, and induces acute emetic effects after consumption [4], [5]. Pigs is one of the most sensitive species with regard to their response to DON-contaminated feed and thus they are the best model for studying the effects of DON intake on the human and animal intestine or immune system (European Food Safety Authority (EFSA) 2004).

The effects of DON-contaminated feed on pigs have been examined in previous studies. When pigs are fed *Fusarium* toxin-contaminated grains, their body weight and feed intake decrease, and their immune systems and internal organs are partially damaged [6], [7]. However, the risk of this contamination has not been adequately addressed in animal feeding husbandry. To date, several strategies have been used to counteract DON in contaminated grains, such as chemical detoxification, biological detoxification and the use of commercial adsorbents. However, these approaches have many challenges in their implementations, such as a lack of substrate-selectivity, a negative effect on grain quality, etc [8].

Arginine-family amino acids play important regulatory functions in swine nutrition, especially in nutrient metabolism and the immune response [9]. Arginine and glutamine are prototypic arginine-family amino acids that have pivotal functions. It has been reported that dietary supplementation with these two amino acids can improve porcine intestinal immunity and growth performance [10], [11]. Even though many studies have focused on the function of amino acids in pig nutrient metabolism, few studies have examined the roles of arginine and glutamine in DON-affected growing pigs. The objective of the present study was to investigate the effects of dietary supplementation with these amino acids on the impairment induced by DON stress and on immune status in growing pigs. Pigs fed with arginine, glutamine and their Arg+Glu, respectively, were challenged with DON; several parameteres were determined, including indices of growth performance, serum biochemical parameters, amino acid transporters, etc. This study may provide a novel approach for alleviating the effects of DON on animal metabolic recovery.

## Materials and Methods

### Preparation of Mouldy Corn

*Fusarium graminearum* isolate R6576 was obtained from the College of Plant Science & Technology of Huazhong Agricultural University, China. This fungus was cultivated on Potato Dextrose Agar (PDA) at 28°C for 7 days of cultivation, aerial mycelia were obtained and inoculated in CMC liquid medium. Medium that contained conidia was extracted with acetonitrile. The number of conidia was determined using a blood-counting chamber. The concentration of conidia was 5×10^5^/ml. Corn was grown at the Institute of Animal Husbandry, Hunan Academy of Agricultural Sciences, Changsha. One month prior to harvest, corn ears at the silking stage were inoculated with media containing conidia. The infected corn was harvested and stored at −20°C. Mouldy kernels collected from the infected ears were ground, passed through a 10-mesh screen and autoclaved at 121°C for 15 min. The resulting ground mouldy corn was determined to contain 6 mg/kg DON.

### Animals and Management

This study was conducted according to the guidelines of the Declaration of Helsinki and all procedures involving animal subjects were approved by the animal welfare committee of the Institute of Subtropical Agriculture, Chinese Academy of Sciences. A total of forty, 60-d-old healthy growing pigs (Landrace×Yorkshire) with a mean body weight of 16.28±1.54 kg were randomly divided into 5 groups (6 replicates/pigs per group): one non-toxin control, one toxin control and 3 amino acid groups in which pigs were fed 1.0% arginine (Arg), 1.0% glutamine (Gln) and 0.5% Arg+0.5% Gln, respectively. Before the animals were challenged with DON, the pigs in the 3 amino acid treatments were fed the corresponding amino acids, respectively, for 7 days while those in non-toxin control and toxic control were fed commercial diet with 1.64% Alanine as isonitrogenous source. The compositions of the commercial feed were listed in Table 1. After this first period, the non-toxin control group was fed the commercial diet while those in the toxin control and in the 3 amino acid treatments were fed DON-contaminated diets. After 21 days of dietary exposure to DON, and immediately after electrical stunning, the pigs were killed for analysis. Body weight and feed consumption were recorded.

**Table 1:** Composition and nutrient level of the diets.

Ingredients	Contents(%)	Nutrient levelsin diets	Contents (%)
Extrusion Corn	60	Digestive energy MJ/kg	14.48
acidifier	0.24	Crude protein	20.90
Additive premix*	0.85	Lysine·HCl	1.48
Glucose	3.2	Met	0.42
Fish meal	2	Thr	0.90
Soybean meal	20	Calcium	0.80
CaHPO4	1.2	Available Phosphorus	0.45
Limestone	1.19	Crude protein	14.48
Soybean oil	2		
Lysine·HCl	0.28		
Threonine	0.04		
Soybean oil	2		

* Premix provided the following per kilogram of the diet: Vitamin A 2000 IU; Vitamin D3 200 IU; Vitamin E 12 IU; Vitamin K 0.5 mg; Vitamin B12 0.016 mg; Vitamin B2 3 mg Vitamin B3 12.5 mg; folic acid 0.3 mg; Vitamin B5 10 mg;Choline chloride 0.5 mg; Vitamin B1 1 mg; Vitamin B6 1.6 mg; Vitamin B7 0.05 mg; Cu 5 mg; Fe 80 mg; Mn 3 mg; Zn 85 mg;I 0.1 mg; Se 0.3 mg.

### Sample Collection

Blood samples were collected and centrifuged at 3000 rpm/min for 10 min. Serum was separated into Eppendorf tubes, and stored at −80°C for further analysis. The liver, spleen, kidney and heart were removed and weighed. The weights were recorded both as the organ weight and the weight as a percent of the total body weight. An intestine sample was cleaned several times in ice-cold phosphate-buffered saline (PBS). Intestinal mucosa was collected using a glass slide.

### Determination of Serum Biochemical Parameters

Assay kits for the analysis of serum biochemical parameters were obtained from Nanjing Jiancheng Biotechnology Company, China. Serum fasting blood glucose (GLU), albumin (ALB), alanine aminotransferase (ALT), aspartate aminotransferase (AST), blood urea nitrogen (BUN), creatinine (CRE), and alkaline phosphatase (ALP) were determined using an Automatic Biochemistry Radiometer (Au640, Olympus).

### Determination of Serum Hormonal Components and Cytokines

Growth hormone (GH), insulin-like growth factor 1 (IGF1), superoxide dismutase (SOD), interleukin (IL)-2, IL-1β and tumour necrosis factor α (TNFα) were measured with the use of ELISA test kits (Beijing Laboratory Biotech Co., LTD, China).

### RNA Extraction and cDNA Synthesis

The intestine tissue sample was pulverized under liquid nitrogen. Total RNA was isolated from 100 mg of the homogenate using the TRIzol reagent (Invitrogen, Carlsbad, CA, USA) and treated with DNase I (Invitrogen) according to the manufacturer’s instructions. The RNA quality was checked by 1% agarose gel electrophoresis, under staining with 10 µg/mL ethidium bromide. The RNA had an OD260:OD280 ratio between 1.8 and 2.0. First-strand cDNA was synthesized with Oligo (dT)20 and Superscript II reverse-transcriptase (Invitrogen).

### Quantification of mRNA by real-time RT-PCR Analysis

Primers for RT-PCR were designed with Primer 5.0 based on the cDNA sequence of porcine Cationic amino acid transporter member1 (s) to produce an amplification product (Table 2). β-actin was used as an internal reference gene to normalize target gene transcript levels. Real-time PCR was performed using SYBR Green PCR Mix, containing MgCl2, dNTP, and Hotstar Taq polymerase. Two µL of cDNA template was added to a total volume of 25µL containing 12.5µL SYBR Green mix, and 1µmol/L each of the forward and reverse primers. We used the following protocol: (i) pre-denaturation (10 s at 95°C); (ii) amplification and quantification, 40 cycles (5 s at 95°C, 20 s at 60°C); and (iii) melting curve (60–99°C with a heating rate of 0.1°C S−1 and fluorescence measurement). The identity of each product was confirmed by dideoxy-mediated chain termination sequencing at Sangon Biotechnology, Inc. We calculated the relative expression ratio ® of mRNA as R = 2−△△Ct. The efficiency of real-time PCR was determined by the amplification of a dilution series of cDNA according to the equation 10(−1/slope), and the results for target mRNA were consistent with those for β-actin. Negative controls were created by replacing cDNA with water.

**Table 2:** Primers used for RT-PCR.

Gene	Primer sequence	Amplicon(bp)	Accession No.
CAT1	Sense 5′- CATCAAAAACTGGCAGCTCA-3′	138 bp	NM_001012613.1
	Antisense 5′- TGGTAGCGATGCAGTCAAAG-3′		
EAAC1	Sense 5′- ATA GAA GTT GAA GAC TGG GAA AT-3′	187 bp	JF521497.1
	Antisense 5′- GTG TTG CTG AAC TGG AGG AG-3′		
β-actin	Sense 5′- GGATGCAGAAGGAGATCACG*-*3′	130 bp	DQ845171
	Antisense 5′- ATCTGCTGGAAGGTGGACAG-3′		

### Statistical Analysis

The data are expressed as means ± SEM. All data were analyzed by ANOVA followed by multiple comparison test using LSD (Least-significant difference) (SAS V6.12). Differences with P values <0.05 were considered to be statistically significant.

## Results

### Growth Performance of pigs fed a DON-contaminated Diet

The cumulative performance results of growing pigs are summarized in Table 3. There was no significant difference between toxin control and amino acid groups with regard to average daily gain (ADG), but this value in non-toxin control was significantly higher than those in the other groups (P<0.05). Since pigs often refused to eat DON-infected food, the average daily feed intake (ADFI) in toxin control was significantly lower than those in the other 4 groups (P<0.05). The non-toxin control showed the highest ADFI (P<0.05). The ADFI in the arginine group was higher than those in the glutamine and Arg+Glu groups. Toxin control showed the lowest ratio of feed to gain (F/G), which was influenced by the refusal of food during the feeding period (P<0.05). F/G in the non-toxin control was slightly lower than those in the 3 amino acid groups.

**Table 3:** Growth performance of growing pigs fed with diets containing DON contaminated corn from 60 day to 88 day (n = 6).

Item	Non-toxin	Toxin control	Arginine	Glutamine	Arg+Glu	P
ADG(g)	421.6±9.84a	378.8±12.76b	370.5±7.83b	364.2±6.28b	372.7±9.21b	<0.01
ADFI(g)	1021.2±17.1a	870.1±19.0d	922.0±13.8b	891.3±16.4cd	915.5±15.8bc	<0.01
F/G	2.43±0.18	2.31±0.24	2.47±0.3	2.45±0.21	2.46±0.13	0.7128

Results are expressed as means ± SEM for six animals. Values within a row sharing different superscript letters differ (P<0.05).

### Relative Organ Weights in Pigs fed a DON-contaminated Diet

Table 4 showed the effects of a DON-contaminated diet on relative organ weights in 60- to 88-day-old pigs. There was no difference in the relative heart weights between non-toxin control and treatment groups (P>0.05). As a result of the detoxifying function of the liver, the relative liver weight in toxin control was 10.66% greater than those in non-toxin control and glutamine groups, respectively. The relative liver weights in the arginine and Arg+Glu groups were also lighter than that in toxin control, but these differences were not significant (P>0.05). There were no significant differences among the groups with regard to the spleen and kidney.

**Table 4:** Relative organ weights (g/kg BW) of growing pigs fed with diets containing DON contaminated corn (n = 6).

Item	Non-toxin	Toxin control	Arginine	Glutamine	Arg+Glu	P
Heart	0.53±0.12	0.50±0.13	0.45±0.05	0.48±0.03	0.51±0.08	0.6131
Liver	2.44±0.13b	2.70±0.23a	2.56±0.21b	2.48±0.20ab	2.55±0.18b	0.0027
Spleen	0.24±0.03	0.19±0.02	0.20±0.04	0.22±0.02	0.23±0.04	0.0581
Kidney	0.45±0.06	0.47±0.04	0.43±0.03	0.46±0.04	0.45±0.03	0.5565

Results are expressed as means ± SEM for six animals.Values within a row sharing different superscript letters differ (P<0.05).

### Serum Biochemical Parameters

Table 5 shows effect of a DON-contaminated diet on serum biochemical parameters of pigs from age 60 to 88 days. While non-toxin control tended to show the highest fasting blood glucose (GLU) level, there were no significant differences among the groups. The blood urea nitrogen (BUN) values in toxin control and Arg+Glu groups were much higher than that in non-toxin control (P<0.001). The arginine group had the highest concentration of creatinine (CRE), and non-toxin control had the lowest concentration. Albumin (ALB) levels in toxin control and arginine groups were 17.7% and 21% less than those in non-toxin control, respectively (P<0.01). The alanine aminotransferase (ALP) activities in toxin control and arginine groups were 26.4% and 16.4% higher than that in non-toxin control, respectively. There was no significant difference in ALP activity among non-toxin control, glutamine and Arg+Glu groups. The ALT activities in toxin control and amino acid groups were significantly greater than that in non-toxin control (P<0.001). While ALT and AST in toxin control were significantly higher than those in non-toxin control (P<0.01), these two values were decreased in the amino acid groups, especially in the arginine and Arg+Glu groups.

**Table 5:** Serum chemical parameters of growing pigs fed with diets containing DON-contaminated corn (n = 6).

Item	Non-toxin	Toxin control	Arginine	Glutamine	Arg+Glu	P
GLU (mmol/L)	7.93±0.94	7.42±0.59	7.85±0.85	7.28±0.93	7.64±0.88	0.6413
BUN (mmol/L)	5.28±0.51a	6.82±0.72b	6.18±0.64ab	6.35±0.7ab	6.61±0.64b	0.0039
ALB(g/L)	35.16±3.46a	28.95±3.21ab	27.78±3.12b	32.72±3.55ab	30.7±4.26ab	0.0101
CRE (µmol/L)	48.23±5.75	53.39±8.70	59.73±6.85	54.81±7.10	51.38±5.29	0.0834
ALP (U/L)	742.3±34.3bc	938.6±98.3a	863.7±64.1ab	790.7±83.4bc	709.3±79.5c	0.001
ALT(U/L)	56.67±7.23a	80.92±7.38b	70.83±6.18b	80.17±6.84b	73.67±7.27b	<0.001
AST(U/L)	93.47±8.94d	133.84±10.63a	115.21±9.38bc	129.76±8.23ab	109.84±8.47c	<0.001

Results are expressed as means ± SEM for six animals.Values within a row sharing different superscript letters differ (P<0.05).

### Serum Hormonal Components and Cytokines

Table 6 shows the effect of dietary treatment with DON on serum IGF1, SOD and GH. The serum activity of insulin-like growth factor 1 (IGF1) in toxin control was significantly lower than those in the other groups, except the arginine group (P<0.05). Toxin control also had the lowest SOD level (P<0.01). The SOD value in the Arg+Glu group was lower than those in non-toxin control and other amino acid groups, while the arginine group had the highest value. Compared with non-toxin control, the treatment groups had a lower expression of growth hormone (GH), and toxin control had the lowest level. IL-2 and TNFα levels in toxin control were significantly higher than those in the other groups (P<0.01). The non-toxin control had the highest IL-1β activity, and there was no difference between the amino acid groups and toxin control (P<0.01).

**Table 6:** Serum hormonal characters and immune relevant cytokines of growing pigs fed with diets containing DON-contaminated corn (n = 6).

Item	Non-toxin	Toxin control	Arginine	Glutamine	Arg+Glu	P
IGF1 (pg/ml)	92.18±2.51a	81.03±2.62b	87.43±1.35a	89.56±4.57a	88.84±3.15a	<0.001
SOD(U/ml)	117.5±6.26a	84.48±8.97c	123.4±5.93a	115.5±9.07ab	103.2±8.29b	<0.001
GH (ng/ml)	29.01±0.88a	25.99±0.64b	27.67±1.52ab	28.35±0.87a	27.86±0.79a	<0.001
IL-2 (pg/ml)	8.24±0.35b	12.9±1.06a	8.68±0.98b	9.29±0.82b	9.13±1.21b	<0.001
TNFα	0.2±0.02b	0.27±0.018a	0.22±0.017b	0.22±0.02b	0.23±0.016b	<0.001
IL-1β	2.47±0.56b	7.79±1.77a	6.72±3.3a	6.08±1.03a	6.19±2.15a	0.0015

Results are expressed as means ± SEM for six animals.Values within a row sharing different superscript letters differ (P<0.05).

### mRNA Expression of Amino Acid Transporters

Two critical intestinal amino acid transporters (AATs; CAT1 and EAAC1) mRNA expressions were tested at the end of feeding. As shown in Table 7, the AAT mRNA expression values in toxin control were significantly lower than those in non-toxin control (P<0.001). The total AAT mRNA levels in non-toxin control were highest among all 5 groups. The pigs in the three amino acid groups had higher levels of EAAC1 than those in non-toxin control and toxin control. Furthermore, the glutamine group had a higher level of CAT1 than the arginine and Arg+Glu groups.

**Table 7:** Intestinal amino acid transporters of growing pigs fed with diets containing Fusarium toxin contaminated corn (n = 6).

Item	Non-toxin	Toxin control	Arginine	Glutamine	Arg+Glu	P
CAT1	1.21±0.15a	0.58±0.06c	0.70±0.09bc	0.78±0.11b	0.72±0.07bc	<0.001
EAAC1	1.00±0.07a	0.41±0.06c	0.81±0.11b	0.79±0.13b	0.77±0.08b	<0.001

Results are expressed as means ± SEM for six animals.Values within a row sharing different superscript letters differ (P<0.05).

## Discussion

Trichothecenes are chemical mycotoxins that are mainly produced by *Fusarium, Trichoderma, Myrothecium,* etc., and have a strong immunosuppressive effect on human and animal health [1]. DON is a member of the trichothecene family, becoming one of the most common mycotoxin contaminant in grains [12]. Most studies have indicated that DON has a harmful effect on animal health or have sought to develop methods for the detoxification of feed materials.However, few investigations have focused on improving an animal’s internal metabolic state through nutrition to counteract the damage done by mycotoxins. In this study, we sought to evaluate the effects of dietary supplementation with amino acids on both the impairment induced by DON stress and immunity in pigs. In the present study, pigs were challenged with the *Fusarium* mycotoxin DON-contaminated (6mg/kg feed) diet for 21 days after an initial 7-day period of amino acid supplementation. Growth performance and relative organ weight were recorded; serum biochemistry, hormonal components and cytokines were analyzed as a reflection of the animal’s condition.

Previous studies have demonstrated a reduction in growth performance when pigs were fed that had been contaminated with different concentrations of DON [13]–[15]. Dersjant-Li et al. (2003) indicated that DON at 600 µg/kg lead to a 5% reduction in the growth of pigs [16]. Döll et al. (2003) tested different amounts of DON (from 0.2 to 3.9 mg/kg) in the diet and found that mean body weight gain decreased from 0.584 to 0.472 kg/d [6]. However, other studies have reported that low levels of DON had no significant effect on body weight gain in pigs [17], [18]. Thus, this issue is still controversial, and may need further investigation. DON concentration used in pigs differed depends on their experiments. Tiemann et al (2008) used 9.57 and 0.358 mg/kg DON/ZON contaminated diet for pregnant sows to investigating changes in the spleen and liver and full-term piglets, and Goyarts et al (2007) studied the carry-over of Fusarium toxin DON (6.68 mg/kg) in pigs [19], [20]. In our study, we chose 6 mg/kg DON in diet for experiments, mainly because the occurrence and contamination of DON in grains was extremely higher in central south region (for example Hunan Province) than in other places of China [21]. Our results showed the consumption of DON by growing pigs decreased ADG by 11.3% (P<0.01). There were no significant differences in ADG between the DON and amino acid groups. Even though the three amino acid groups were fed amino acids for 7 days at first period, these amino acids did not appear to influence body weight gain compared with the DON group. This may be at least partly due to the roles of amino acids in animals fed a DON-contaminated diet; these nutrients may play important roles in alleviating the impairment of metabolism and improving immunity homeostasis. Many investigators have indicated that dietary supplementation with arginine or glutamine can improve the growth performance of pigs [22]–[25]. The consumption of diet contaminated with DON would reduce this improvement. Indeed, ADFI in toxin control was significantly lower than that in non-toxin control, as well as the arginine and Arg+Glu groups (P<0.01). The feed/gain (F/G) ratio in toxin control tended to be lower than those in other groups, but no significant differences were observed among any of the groups. These results show that amino acids notably alleviated food-refusal in pigs. However, the efficiency of feed utilization is not useful for comparing DON-free and DON-treated pigs, since there were no significant differences in the F/G ratios among these groups. Similar results were reported in studies elsewhere [26], [27].

In the present study, there were no significant differences in the relative organ weights of growing pigs in the five groups. Rotter et al. (1994) reported that there were no changes in organ weights with the use of DON concentrations ranging from 750 to 3000 µg/kg [28]. Chaytor et al. (2011) analyzed the effect of the combination of DON and aflatoxins on the weights of internal organs, and found no changes in the weights of the liver, kidney or spleen [29]. In our study, the liver weight in toxin control was 10.7% greater than that in non-toxin control. This increase may be due to the edema and enlargement of the liver caused by DON toxicity. An extended period (7 weeks) of DON contamination, as well as a higher DON concentration (more than 3900 µg/kg), have been reported to be important factors for increasing the weights of the liver and kidney [30]. Therefore, the DON concentration and exposure time likely contribute to the relative organ weights in DON-contaminated pigs. The weights of the liver, spleen and kidney in the amino acid groups were not significantly different from those in non-toxin control and toxin control, which mean that an increase in protein synthesis by amino acid intake and organ enlargement caused by severe systemic toxicity may competitively interact to influence relative organ weights.

Serum levels of BUN, ALB, GLU, CRE, AST, ALT and ALP were tested as a reflection of the metabolism and inner organ status of pigs. ALB was increased in the glutamine and Arg+Glu groups compared with toxin control and arginine group, but this difference was not significant. Decreased albumin levels have been found in pigs fed a DON-contaminated diet [13], [31]. However, other studies have reported that dietary exposure to DON has no significant effect on plasma protein concentrations (total protein, albumin and fibrinogen) [32]. Alkaline phosphatase (ALP) is secreted by the mucosal cells that line the bile system of the liver, and thus is indicative of liver damage. The increase in ALP in toxin control reflected the abnormal excretion of liver metabolites due to DON-induced systemic toxicity, as described in a previous study [29]. Dietary supplementation with amino acids, especially glutamine and the combination of arginine and glutamine, decreased the ALP levels in pigs, and thus clearly reduced liver injury and enhanced the detoxification function of the liver. Serum AST and ALT levels have been reported to be sensitive indicators of liver injury, since an increase in these values reflects leakage from injured hepatocytes [33]. The decrease in AST in the arginine and Arg+Glu groups compared with toxin control indicated that this mechanism is improved by amino acid intake.

In trichothecene group, DON and Nivalenol (NIV), as well as T-2 toxin, were more harmful mycotoxins in pig than others [34]–[36]. Moreover, pigs are considered the most susceptible farm animal to trichothecenes, including DON [32]. Previous studies had investigated the effect of DON on intestine and immune system of mammals. Marzocco et al. and Bianco et al studied the effect of NIV and DON on rat intestinal cells, showing that both NIV and DON stimulate apoptosis through apoptotic pathway involves ERK, pro-apoptotic protein Bax, caspase-3, etc [37], [38]. Awad et al and Pinton et al also reported that DON would induce cytotoxicity and metabolic stress in IPEC cells [39], [40]. Exposure of pigs to DON has been associated with a reduction of feed intake, disturbances in immunological function and inflammation of the intestinal tract [41], [42]. The inhibition of protein synthesis following exposure to DON causes the brain to increase the uptake of tryptophan and the synthesis of serotonin, which is responsible for the anorexic effects of DON [43]. Arginine and glutamine play crucial roles in swine nutrition metabolism, such as fat synthesis and oxidation, nutrient transport, and ammonia detoxification. Arginine is well known for its regulatory role in the interorgan metabolism of energy substrates and the function of multiple organs [10]. Meanwhile, glutamine is an important active substance in the body, which can scavenge free radicals and detoxify [44]. Dietary supplementation with arginine or glutamine has been widely used to enhance growth performance and prevent intestinal injury in swine production [45], [46]. Therefore, we chose these two amino acids to investigate the effects of supplementation with amino acids on DON-induced impairment and immunity in DON-contaminated growing pigs. We measured several related hormonal parameters and pro-inflammatory cytokines in our experiment, which are associated with the porcine immune status, such as IL-1β, TNFα, and IL-2 [47]. Li et al. (2012) reported that dietary supplementation with arginine reduced liver morphological impairment by decreasing the release of pro-inflammatory cytokines in the liver [48]. Additionally, other studies have shown that arginine plays a protective effect in models of liver injury, such as hepatic ischemia reperfusion injury in pigs and acute cholestasis-induced liver damage in rats [49], [50]. In our research, IL-2 and TNFα values in the amino acid groups were significantly lower than those in toxin control (P<0.01) and there were no significant differences among non-toxin control and amino acid groups. Even though the IL-1β values in the amino acid groups were not significantly different from that in toxin control, a slight decrease was observed in pigs that had been nutritional prevented with amino acids. These results suggest that amino acids have a protective effect in the immune system and reduce injury to the liver in DON-contaminated pigs. The results in toxin control were consistent with those in previous studies. Zhou et al. (1998) reported that supplementation with DON increased the mRNA expression of IL-2 and TNFα [51]. These cytokines have been reported to be associated with the enhanced differentiation of IgA-secreting B cells. In the present study, IGF-1, SOD and GH in toxin control were markedly decreased compared to those in non-toxin control (P<0.01), while amino acid intake reduced this trend. Indeed, arginine has been reported to act as a potent stimulator of insulin and growth hormone secretion [52], [53].

The small intestine of pigs has been reported to use exogenous glutamine to produce citrulline, which is converted locally into arginine in the gut of neonatal pigs. Approximately 40% of arginine is catabolised in the first pass through the small intestine [9]. To gain insight into the status of amino acid utilization in porcine intestine, we examined the mRNA expression of two amino acid transporters. Cationic amino acid transporter member1 (CAT1) plays a major role in the intestinal transport of cationic amino acids, such as L-lysine and L-arginine [54]. Excitatory amino acid transporter 1 (EAAC1) is a sodium-dependent glutamate transporter that can transport neutral amino acids, especially glutamate and cysteine, into intestinal vesicles [55]. The values for CAT1 mRNA expression in the amino acid groups, especially in the glutamine and Arg+Glu groups, were higher than that in toxin control. The values for EAAC1 mRNA expression in the amino acid groups were significantly higher than that in toxin control (p<0.01). Dietary supplementation with amino acids clearly enhanced the expression of the corresponding amino acid transporters in porcine intestine. However, the mRNA expression of amino acid transporters in the amino acid groups did not strictly correspond to the changes in amino acid intake. This relationship between amino acid expression and amino acid intake in DON-contaminated pigs will require further investigation.

Based on previous studies, toxicity and damage of DON on animal mechanism were mainly through mitogen-activated protein kinases (MAPKs) signal pathways, via the “ribotoxic stress response”, binding to ribosomes and promote MAPKs phosphorylation, which correlated with protein synthesis inhibition and precedes apoptosis [56]. But in vitro and in vivo investigations showed that glutamine and arginine decrease the proinflammatory cytokine production and preserve from the impairment induced by LPS in associated with changes of p38 MAPKs and nuclear factor-*κ*B (NF-κB) pathways [57]–[59]. Therefore, for the mechanism of arginine and glutamine alleviating the impairment induced by deoxynivalenol stress and improving immune relevant cytokines, we hypothesis that exogenous arginine and glutamine increasing would improve animals metabolism involved MAPKs and NF-κB pathways, further studies were needed.

In conclusion, in DON-contaminated growing pigs, dietary supplementation with amino acids significantly reduced anorexic effects during the feeding period. Simultaneously, an enhanced utilization of amino acids in pigs alleviated the impairment induced by DON stress and immune relevant cytokines in growing pigs. Improved nutrition may be a novel approach for mitigating mycotoxin contaminations in animal production.
